# *Bacillus cereus* Group Bacteriophage Flapjack Genome Sequence

**DOI:** 10.1128/genomeA.00700-17

**Published:** 2017-08-03

**Authors:** Ivan Erill, Steven M. Caruso

**Affiliations:** Department of Biological Sciences, University of Maryland, Baltimore County, Baltimore, Maryland, USA

## Abstract

The *Bacillus cereus* group bacteriophage Flapjack, a double-stranded DNA (dsDNA) *Myoviridae* isolate collected from soil collected in Washington, DC, is a member of cluster C3 and encodes an intramolecular chaperone-containing tail fiber protein previously found in *Podoviridae* and *Siphoviridae* but not annotated in the *Myoviridae*.

## GENOME ANNOUNCEMENT

The *Myoviridae* bacteriophage Flapjack was isolated from a soil sample collected in Washington, DC, USA (38°57′04.0″N, 77°04′51.0″W), and cultivated using *Bacillus thuringiensis* subsp. *kurstaki* (ATCC 33679) as a host by UMBC Phage Hunters as part of the 2016–2017 SEA-PHAGES course (1). Sequencing was performed by the Pittsburgh Bacteriophage Institute to approximately 485-fold coverage by Illumina sequencing. *Bacillus* phage Flapjack has a linear double-stranded DNA chromosome that is 166,137 bp in length, with direct terminal repeats that are 2,806 bp in length and a G+C content of 37.6%. Genome ends were determined by examination of Illumina sequencing reads in Consed. Flapjack’s chromosome contains 288 protein-coding genes and has no identified noncoding RNA (ncRNA) genes. Of the predicted start codons, 80.9% are AUG, 7.6% are GUG, and 11.5% are UUG. Flapjack has an average (±SD) nucleotide identity of 87% ± 5% with phages belonging in the C3 subcluster (2, 3) and shows particularly high sequence similarity to *Bacillus* phages Spock, Typhen, and GypsyDanger. This, together with whole-genome comparison (4) and phylogenetic analysis (5), confirms Flapjack as a member of subcluster C3.

Transmission electron microscopy examination revealed that Flapjack has an icosahedral head with a width of approximately 91 nm and a contractile tail length of 232 nm. Additional information about Flapjack, Typhen, GypsyDanger, and other *Bacillus* phages isolated by undergraduate researchers of the 2016–2017 course and previous cohorts can be found in the *Bacillus* Phage Database (http://bacillus.phagesdb.org/).

*B. thuringiensis* subsp. *kurstaki* is a member of the *Bacillus cereus* group, which includes Gram-positive, rod-shaped, spore-forming species, including *B. anthracis*, *B. cereus*, *B. thuringiensis*, and others (6). Flapjack demonstrated a fairly limited host range among *B. cereus* group hosts and was able to infect just half of those tested: *B. thuringiensis* subsp. *kurstaki* and Al Hakam,* B. cereus* Frankland and Frankland (ATCC 14579), *B. cereus* Gibson 971, and *B. anthracis* delta Sterne. Overall, Flapjack’s host diversity was smaller than that of 65% of the other phages from the same 2016–2017 cohort (*n* = 60).

Flapjack and other examined cluster C phages encode an intramolecular chaperone-containing tail fiber protein showing high similarity to the one found in *Escherichia coli* phage K1F (7) and the intramolecular chaperone domain of the *Enterobacteria* phage T5 L-shaped tail fiber (8). The chaperone domain shows a substantial degree of sequence divergence compared to the tail fiber harboring it and other structural proteins. In some subcluster C4 phages (e.g., *Bacillus* phage Eldridge or *Bacillus* phage Moonbeam), the sequence encompassing the chaperone domain matches instead the PF05895 domain of unknown function, known to be associated with tail fiber genes (9). This intramolecular chaperone has been reported in the in *Podoviridae* and *Siphoviridae*, but it had not previously been annotated in the *Myoviridae*.

### Accession number(s).

The complete genome sequence of *Bacillus* phage Flapjack is available in GenBank with the accession number KY888882.
